# Threats to social safety and neuro-inflammatory mechanisms underlying sexual orientation disparities in depression symptom severity: A prospective cohort study of young adults

**DOI:** 10.1016/j.bbi.2024.03.036

**Published:** 2024-03-27

**Authors:** Richard Bränström, Mark L. Hatzenbuehler, Micah R. Lattanner, Nathan L. Hollinsaid, Thomas W. McDade, John E. Pachankis

**Affiliations:** a Division of Psychology, Department of Clinical Neuroscience, Karolinska Institutet, Stockholm, Sweden; b Department of Psychology, Harvard University, Cambridge, MA, USA; c Department of Public Health, Santa Clara University, Santa Clara, CA USA; d Department of Anthropology and Institute for Policy Research, Northwestern University, Evanston, IL, USA; e Department of Social and Behavioral Sciences, Yale School of Public Health, New Haven, CT, USA

**Keywords:** Early adversity, Social safety, C-reactive protein, Interleukin-6, Tumor necrosis factor alpha, Sexual minorities, Depression

## Abstract

Sexual minority individuals have a markedly elevated risk of depression compared to heterosexuals. We examined early threats to social safety and chronically elevated inflammation as mechanisms contributing to this disparity in depression symptoms, and compared the relative strength of the co-occurrence between chronic inflammation and depression symptoms for sexual minorities versus heterosexuals. To do so, we analyzed data from a prospective cohort of sexual minority and heterosexual young adults (*n* = 595), recruited from a nationally representative sample, that included assessments of early threats to social safety in the form of adverse childhood interpersonal events, three biomarkers of inflammation (i.e., CRP, IL-6, TNF-α) measured at two time points, and depression symptoms over four years. In pre-registered analyses, we found that sexual minorities experienced more adverse childhood interpersonal events, were more likely to display chronically elevated inflammation, and reported more severe depression symptoms than heterosexuals. Adverse childhood interpersonal events and chronically elevated inflammation explained approximately 23 % of the total effect of the association between sexual orientation and depression symptom severity. Further, there was an increased coupling of chronically elevated inflammation and depression symptoms among sexual minorities compared to heterosexuals. These results provide novel longitudinal, population-based evidence for the role of chronically elevated inflammation in linking threats to social safety during childhood with depression symptom severity in young adulthood, consistent with the primary tenets of the social signal transduction theory of depression. Our study extends this theory to the population level by finding that members of a stigmatized population (i.e., sexual minorities) experience a greater risk of depression because of their greater exposure to adverse childhood interpersonal events and the subsequent link to chronic inflammation, highlighting potential biopsychosocial intervention targets.

## 1. Introduction

Sexual minority individuals have a markedly elevated risk of depression compared to heterosexuals (Bostwick et al., 2010; Bränström et al., 2018; Caceres et al., 2019; Hughes et al., 2010; Plöderl and Tremblay, 2015; Semlyen et al., 2016), motivating the search for biopsychosocial mechanisms that underlie this disparity. The *social signal transduction theory of depression* proposes that threats to fundamental needs for social relatedness, such as adverse interpersonal events (e.g., abuse, social exclusion), are more likely to impact psychological, biological, and clinical outcomes than other threats (Slavich and Sacher, 2019; Slavich and Irwin, 2014; Slavich, 2020). Exposure to these interpersonal threats can begin early in life (McLaughlin et al., 2012), with links to subsequent inflammation (Hatzenbuehler et al., 2014; Hostinar et al., 2018), which may in turn predict elevated depression symptom severity and persistence in some individuals, particularly in the context of other risk factors, such as prior depression or other stressors (Chu et al., 2019; de la Torre-Luque et al., 2019; Zalli et al., 2016; Hamer et al., 2009; Khandaker et al., 2014). The theory further suggests that early interpersonal threats can galvanize the neural link between the brain and the inflammatory system—a process described as *neuro-inflammatory sensitization* (Slavich and Irwin, 2014)—to increase individuals’ preparation for threat and their risk of depression in expectation of future threats.

Scholars have recently applied the social signal transduction theory of depression to the context of sexual minority stigma, arguing that sexual minorities’ disproportionate exposure to threats to social safety—including bullying (Fedewa and Ahn, 2011), discrimination (Rice et al., 2021), and family rejection (Hatzenbuehler and Pachankis, 2016)—results in elevations in inflammation that could explain their increased risk of depression relative to heterosexuals (Diamond et al., 2021; Diamond and Alley, 2022; Slavich et al., 2023). In initial support of this hypothesis, studies have identified a link between exposure to discrimination and inflammation among sexual minority individuals (Cook et al., 2022; Wardecker et al., 2021) and other stigmatized groups (Cuevas et al., 2020). However, whether inflammatory processes mediate the association between stigma exposure and sexual minority individuals’ mental health problems, including depression, remains untested (Diamond et al., 2021). The current study begins to address this gap by exploring whether chronic inflammation linked to childhood exposure to adverse interpersonal events can explain sexual orientation disparities in depression symptoms during young adulthood, a potentially sensitive period for the development of internalizing psychopathology (Hankin et al., 1998; Twenge et al., 2019). To do so, we took advantage of a prospective cohort of sexual minority and heterosexual young adults, recruited from a nationally representative sample, that included assessments of (1) depression symptoms over four years, (2) inflammation at two time points, and (3) early exposure to threats to social safety (i.e., adverse childhood interpersonal events).

We made the following three pre-registered predictions. First, in line with the social signal transduction theory of depression (Slavich, 2020), we hypothesized that early threats to social safety in the form of adverse childhood interpersonal events would prospectively predict depression symptoms in young adulthood and that this association would be mediated by chronically elevated inflammation. Second, we hypothesized that sexual minority individuals, compared to heterosexuals, would report greater exposure to adverse childhood interpersonal events and thus would be more likely to experience chronically elevated inflammation (Diamond et al., 2021); further, we hypothesized that these early threats to social safety and chronic inflammation would independently and serially mediate the longitudinal relationship between sexual orientation and depression symptoms. Third, because sexual minorities (vs. heterosexuals) were expected to be at increased risk of exposure to various forms of threats to social safety, including adverse interpersonal events during childhood, we hypothesized that the association between chronically elevated inflammation and depression symptoms would be stronger among sexual minorities compared to their heterosexual peers. This finding would provide evidence of an increased biological coupling of chronically elevated inflammation and depression symptoms among sexual minorities, consistent with the neuro-inflammatory sensitization hypothesis (Slavich and Irwin, 2014).

## 2. Methods and materials

### 2.1. Participants

Data came from four annual waves of the Pathways to Longitudinally Understanding Stress (PLUS) study, a longitudinal population-based cohort study of young adults in Sweden initiated in 2019. The cohort was recruited from the Swedish National Public Health Survey (SNPHS), a randomly selected, nationally representative sample. In the 2015, 2016, and 2018 SNPHS data collections, one question regarding sexual orientation was included, to which most participants (96 %) responded.

All 2,973 young adult participants (ages 18–34) who identified as non-heterosexual in the SNPHS were invited to the PLUS cohort, along with an equal-sized randomly selected sample of heterosexual participants ages 18–34. A total of 2,222 (37.8 % of those invited) provided consent and completed the Wave 1 survey (October 2019). Among those, all sexual minority participants consenting to participate (*n* = 826) and an equal-sized sample of heterosexual respondents were invited to collect blood at home and submit these samples for storage and analysis at Wave 1 and again two years later at Wave 3. 1,084 individuals (65.8 % of invited sexual minorities; 65.7 % of invited heterosexuals) returned biomarker samples at Wave 1, and 661 individuals (56.9 % of sexual minorities and 52.5 % of heterosexuals returning the Wave 1 biomarker samples) returned Wave 3 biomarker samples. Our final analytic sample includes all individuals with biomarker data from both waves (*n* = 595). We found no significant differences between retained and attritted participants in Wave 1 depression symptoms, inflammation, or sexual orientation. The timing of each assessment is illustrated in Supplementary Figure S1. The Stockholm Regional Ethical Review Board (2018/1517–31) approved procedures. The study protocol was preregistered at: https://osf.io/4basq.

### 2.2. Self-report measures

#### Sexual orientation.

A series of questions at each wave were used to assess sexual orientation (Bränström et al., 2023). Participants were classified as sexual minorities if they reported identifying as lesbian, gay, bisexual, queer, pansexual, asexual, or demisexual, or if they otherwise confirmed that they were comfortable being referred to as LGBTQ + at any of the four waves of assessment.

#### Adverse childhood interpersonal events.

Nine items from the adult retrospective version of the Juvenile Victimization Questionnaire assessed early threats to social safety in the form of adverse childhood interpersonal events before age 18 years, including exposure to physical assault, sexual assault, maltreatment, and peer and sibling victimization (Finkelhor et al., 2011). The scale has demonstrated adequate psychometric properties in previous studies, including good internal consistency, test–retest reliability, and construct validity (Mathews et al., 2023; Meinck et al., 2022; Finkelhor et al., 2005). A sum score was calculated (range = 0–9; M = 2.4, SD = 2.0).

#### Depression symptoms.

Depression symptoms were assessed at Waves 1 through 4 using the 20-item Center for Epidemiologic Studies Depression (CES-D) scale (Radloff, 1977), which measures the frequency of depression symptoms during the past week on a 4-point scale. Responses were summed, with a range from 0 to 60; higher scores indicate more frequent depression symptoms (Cronbach’s α_Wave 1_ = 0.92, α_Wave 2_ = 0.91, α_Wave 3_ = 0.91, and α_Wave 4_ = 0.92). Participants’ depression symptoms were highly consistent across the four waves (intraclass correlation: 0.88).

#### Covariates.

Sociodemographic characteristics were assessed at Wave 1, including age, sex assigned at birth, gender identity, education, and Swedish nativity. Age, sex assigned at birth, and education were included as covariates in multivariable analyses given their association with sexual orientation and/or depression symptoms (Bränström et al., 2023; Barnes et al., 2014; Bränström, 2017). In the main analyses, several additional variables with potential influence on inflammatory biomarkers were also included as covariates: symptoms of ongoing infection at time of data collection; current use of anti-inflammatory medication; current use of contraceptives; and pregnancy.

We also controlled for tobacco use and weight status (defined as a body mass index [based on self-reported weight and height] of higher or lower than 25); both influence inflammatory biomarkers and have a potential link to social stress exposure and depression symptoms (Chae et al., 2022; Yanbaeva et al., 2007). Because these two variables could therefore lie along the causal pathway, they were only controlled for in sensitivity analyses and were not included in our main analyses in line with recommendations for enhancing causal inference in immunological research (Moriarity et al., 2023).

### 2.3. Biomarkers of inflammation

#### Inflammatory biomarkers.

Three measures of baseline levels of inflammatory activity from two time points were used to construct a latent variable of chronically elevated inflammation: C-reactive protein (CRP), interleukin-6 (IL-6), and tumor necrosis factor alpha (TNF-α). Samples were collected via the at-home dried blood spot (DBS) technique (McDade et al., 2020; Roberts et al., 2016). We sent a self-collection kit (no. 903; Whatman, GE Healthcare Bio-Science Corporation) by postal mail at Waves 1 (November 2019) and 3 (November 2021) with pre-paid return. The samples were stored at Karolinska Institutet Biobank before being shipped to Northwestern University for assaying.

CRP was quantified using a high sensitivity enzyme immunoassay previously validated for DBS samples (McDade et al., 2004). The lower limit of detection (LLD) of the assay is < 0.03 mg/L. Similarly, IL-6 and TNF-α were measured with a multiplex electrochemiluminescent immunoassay protocol specifically validated for DBS samples (McDade et al., 2021). The LLD for IL-6 and TNF-α is 0.4 and 0.9 pg/mL, respectively. Assays were performed in duplicate. The average intra-assay coefficient of variation at Wave 1 was 4.0 % for CRP, 12.7 % for IL-6, and 5.0 % for TNF-α and at Wave 3 was 2.9 % for CRP, 10.6 % for IL-6, and 5.1 % for TNF-α.

#### Measure of chronically elevated inflammation.

We indexed chronically elevated inflammation as follows. First, we transformed CRP, IL-6, and TNF-α values into quartiles so that each participant received a score from 0 to 3 for each biomarker at Waves 1 and 3 depending on the level of each respective biomarker in the sample distribution at that assessment point. Second, to capture the stability of CRP, IL-6, and TNF-α levels at Waves 1 and 3, we assigned each biomarker a score from 0 to 6 based on its quartile rank at each assessment point. Scores ranged from 0 for participants with values in the lower quartile at both assessment points to 6 for participants with values in the upper quartile at both assessment points. Third, these scores were used as indicators in a structural equation model assessing a latent variable of chronically elevated inflammation. All three indicators loaded significantly onto the latent variable, which we preregistered as the primary measure of chronic inflammation for our analyses. Similar composite indicators have been used in longitudinal studies of inflammation and health (Harris et al., 1999; Giovannini et al., 2011; Jylhä et al., 2007). This approach provides a better indicator of chronically elevated inflammation than does a single measure (Del Giudice and Gangestad, 2018) and reduces the risk of type 1 error associated with multiple tests across individual markers.

### 2.4. Statistical analysis

We examined sexual orientation differences in demographic characteristics, adverse childhood interpersonal events, individual inflammatory biomarkers, chronically elevated inflammation, and depression symptom severity using Student’s *t*-test.

To test our first hypothesis, we calculated the indirect effect of chronically elevated inflammation on the longitudinal association between adverse childhood interpersonal events and depression symptoms. To evaluate our second hypothesis, we calculated the indirect effects of adverse childhood interpersonal events and chronically elevated inflammation, both independently and serially, on the association between sexual orientation and depression symptoms. In testing these two hypotheses, depression symptoms across the four waves were examined in two ways using structural equation modelling. First, we created a latent variable with depression severity at each of the four waves as indicators, providing an assessment of the average level of depression symptoms over the four-year period. Second, to capture change in depression symptoms over time, we used a latent growth model, specifying both a latent depression intercept (i.e., the average level of depression symptoms at Wave 1) and slope (i.e., the average change in depression symptoms from Wave 1 to Wave 4).

Third, to test our final hypothesis, we examined sexual orientation as a moderator in structural equational models estimating (a) the association between chronically elevated inflammation and depression symptoms and (b) the indirect effect of chronically elevated inflammation on the association between adverse childhood interpersonal events and depression symptoms.

Analyses were conducted with SPSS version 26 and Mplus version 8. Maximum likelihood was used to estimate direct and indirect effects, and bootstrapped confidence intervals were calculated to assess significance of the indirect effects taking into account possible non-normality of the effect distributions.

## 3. Results

### 3.1. Descriptive statistics

Heterosexuals were somewhat older, had higher education, and were more likely to report a birth-assigned sex of male compared to sexual minorities (Table 1). Correlations between study variables are presented in Supplementary Table S1.

### 3.2. Sexual orientation disparities in depression symptoms, adverse childhood interpersonal events, and inflammation

Sexual minorities reported significantly more severe depression symptoms at all waves and reported exposure to a greater number of adverse childhood interpersonal events than heterosexuals (Table 2). Sexual orientation differences in individual inflammatory biomarkers were small and non-significant at both time points. However, sexual minorities had significantly higher chronically elevated inflammation (i.e., when modeled as a latent variable using all three biomarkers) compared to heterosexuals, adjusting for sociodemographics, medication use, and ongoing infection (0.104, 95 % confidence interval [CI]: 0.005, 0.204).

### 3.3. Chronically elevated inflammation as a mediator of the association between adverse childhood interpersonal events and depression symptom severity

**Figure 1.**
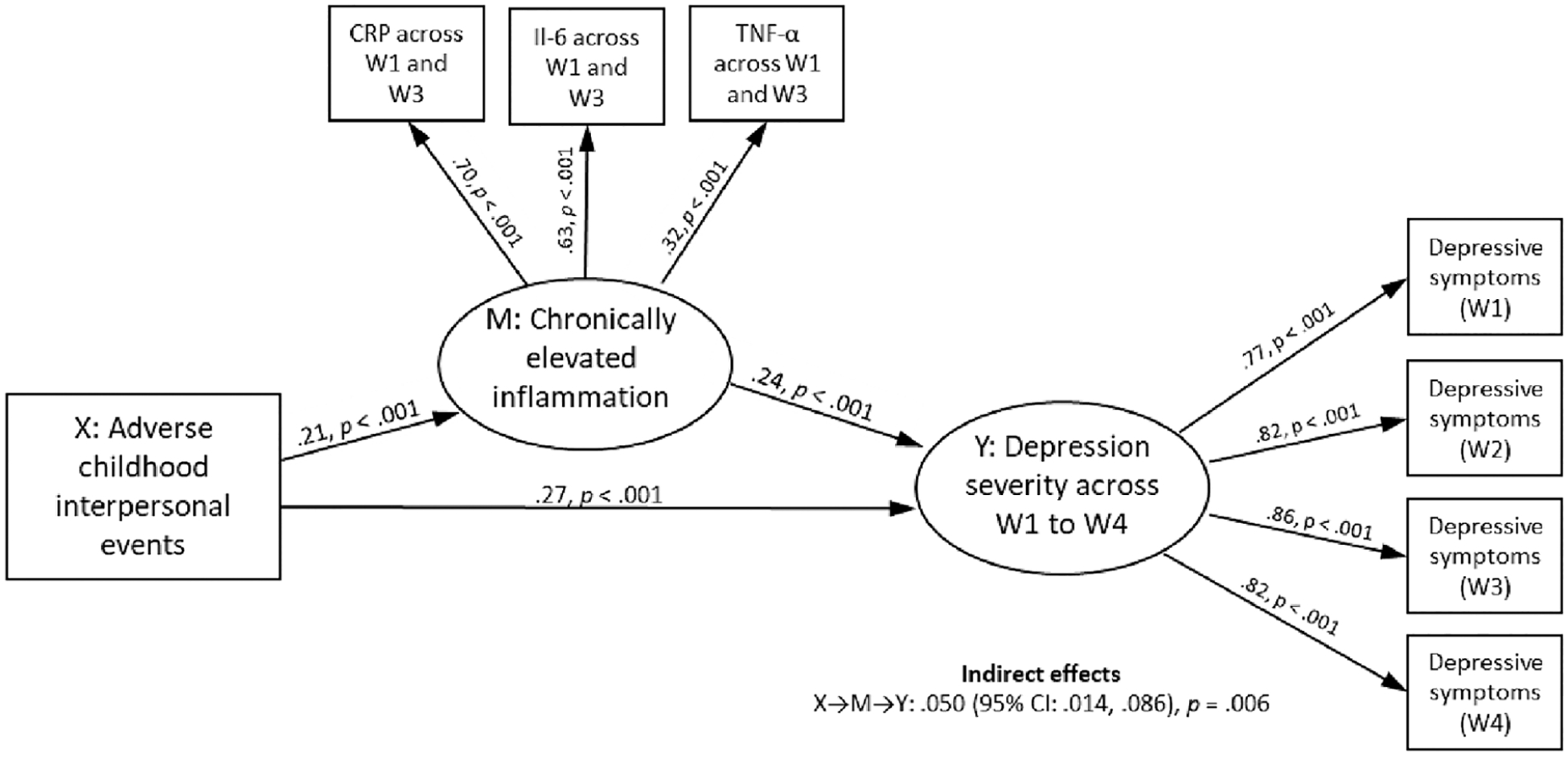
Chronically elevated inflammation as a mediator of the longitudinal association between experiences of adverse childhood interpersonal events and depression symptoms (Supplementary Table S2 presents the full set of estimates including covariates). **Note:** Standardized estimates presented next to model paths. Model fit indices were: χ^2^ (78, N = 595) = 139.71, p < .001; comparative fit index = .96; root mean square error of approximation (90% confidence interval) = .04 (.03 – .05); standardized root mean square residual = .02. The model was adjusted for age, sex assigned at birth, sexual orientation, education, ongoing infection, use of antidepressants and contraceptives, and current pregnancy. CRP=C-reactive protein. Il-6=interleukin-6. TNF-α=tumor necrosis factor alpha.

When modelling depression symptom severity using a latent variable with depression severity at each of the four waves as indicators, we observed a significant indirect effect of chronically elevated inflammation on the association between adverse childhood interpersonal events and depression symptom severity in the full sample (0.050, 95 % CI: 0.014, 0.086; *p* = 0.006; Figure 1, Supplementary Table S2). The indirect effect of chronically elevated inflammation explained 16 % of the total effect of adverse childhood interpersonal events on depression symptom severity across four years of young adulthood.

We found no evidence that chronically elevated inflammation mediated the association between adverse childhood interpersonal events and change in depression symptom severity from Wave 1 to Wave 4 when using a latent growth model (Supplementary Figure S2); thus, we only included a latent variable representing average depression symptom severity over this four-year period in subsequent versions of this mediation model.

### 3.4. Adverse childhood interpersonal events and chronically elevated inflammation as mediators of the association between sexual orientation and depression symptom severity

**Figure 2.**
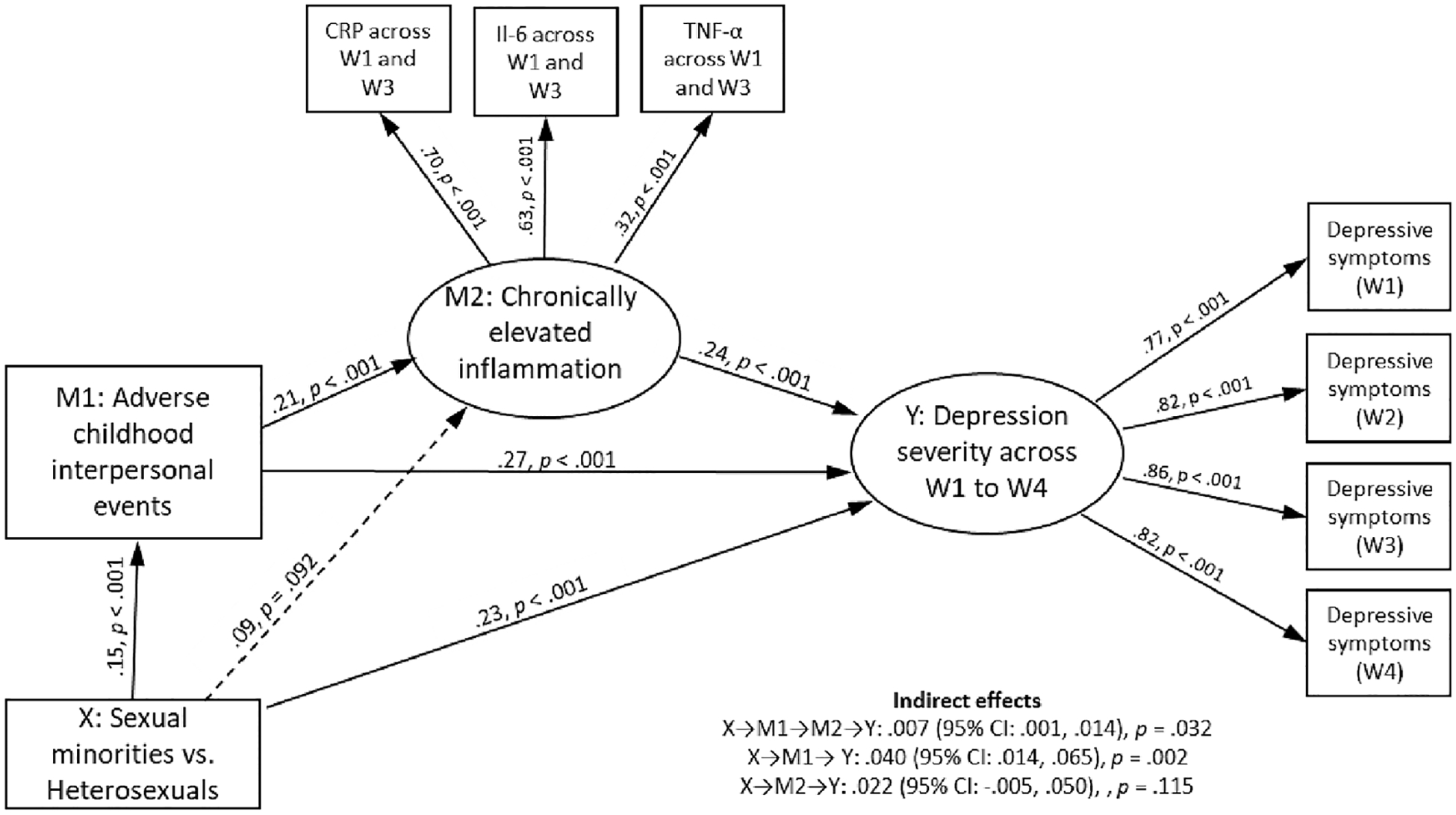
Adverse childhood interpersonal events and chronically elevated inflammation as mediators of the longitudinal association between sexual orientation and depression symptoms (Supplementary Table S3 presents the full set of estimates including covariates). **Note:** Standardized estimates presented next to model paths. Model fit indices were: χ^2^ (78, N = 595) = 139.71, p < .001; comparative fit index = .96; root mean square error of approximation (90% confidence interval) = .04 (.03 – .05); standardized root mean square residual = .02. The model was adjusted for age, sex assigned at birth, education, ongoing infection, use of antidepressants and contraceptives, and current pregnancy. CRP=C-reactive protein. Il-6=interleukin-6. TNF-α=tumor necrosis factor alpha.

We next tested adverse childhood interpersonal events and chronically elevated inflammation as independent and serial mediators of the association between sexual orientation and average depression symptom severity. Whereas adverse childhood interpersonal events independently mediated the longitudinal association between sexual orientation and depression symptom severity (0.040, 95 % CI: 0.014, 0.065, *p* = 0.002; Figure 2 and Supplementary Table S3), chronically elevated inflammation did not (0.022, 95 % CI: −0.005, 0.050, *p* = 0.115; Figure 2 and Supplementary Table S3). However, the association between sexual orientation and depression symptom severity was serially mediated through both adverse childhood interpersonal events and chronically elevated inflammation (0.007, 95 % CI: 0.001, 0.014, *p* = 0.032; Figure 2 and Supplementary Table S3). Together, these two mediators explained 23 % of the total association between sexual orientation and depression symptom severity (adverse childhood interpersonal events [13.4 %], chronically elevated inflammation [7.3 %], and serially through both [2.3 %]).

When modelling depression symptom severity using a latent growth model, we found neither a sexual orientation difference in change in depression symptoms severity from Wave 1 to Wave 4 nor evidence that adverse childhood interpersonal events or chronically elevated inflammation mediated this relationship (Supplementary Figure S3). Thus, we only included a latent variable representing average depression symptom severity over this four-year period in subsequent versions of this mediation model.

### 3.5. Increased coupling of chronically elevated inflammation and depression symptom severity among sexual minorities vs. heterosexuals

To test sexual orientation as a moderator of the association between chronically elevated inflammation and depression symptoms, we conducted a moderated structural equation model with a latent variable for depression severity across all four waves of assessment. This test was statistically significant (0.112; 95 % CI: 0.010, 0.213; *p* = 0.031), providing support for a difference in the association between chronically elevated inflammation and depression symptom severity among sexual minorities compared to heterosexuals. Analyses stratified by sexual orientation showed that the association between chronically elevated inflammation and depression symptom severity was stronger among sexual minorities (0.366; 95 % CI: 0.231, 0.500; *p* < 0.001) than heterosexuals (0.223; 95 % CI: 0.004, 0.442; *p* = 0.046). To determine whether this increased coupling of chronically elevated inflammation and depression symptom severity among sexual minorities was explained by their greater exposure to adverse childhood interpersonal events (relative to heterosexuals), we next examined whether sexual orientation moderated the full mediation model (i.e., the indirect effect of chronically elevated inflammation on the association between adverse childhood interpersonal events and depression symptoms). Although the indirect effect of chronically elevated inflammation was stronger among sexual minorities (0.064; 95 % CI: 0.014, 0.113; *p* = 0.012) than among heterosexuals (0.038; 95 % CI: −0.025, 0.100; *p* = 0.235), a multi-group mediation model found no statistically significant difference between the two groups.

### 3.6. Sensitivity analyses

We conducted several sensitivity analyses not included in our pre-registration. First, we re-ran the mediation models adjusting for covariates with a known link to inflammation (i.e., weight status, tobacco use). These analyses showed a very similar pattern of results to the primary analyses (Supplementary Figure S4–S5).

Second, to compare the magnitude of the indirect effects of adverse childhood interpersonal events on depression symptom severity through each of the three inflammatory biomarkers, we re-ran mediation models separately for each biomarker. Chronic elevations in IL-6 (0.029; 95 % CI: 0.009, 0.049; *p* = 0.005) and CRP (0.017; 95 % CI: 0.001, 0.033; *p* = 0.038), but not in TNF-α (0.005; 95 % CI: −0.005, 0.016; *p* = 0.310), were significant mediators of this association (Supplementary Figure S6a–c). In models including both adverse childhood interpersonal events and separate indicators of chronically elevated inflammation as serial mediators of the association between sexual orientation and depression symptom severity (Supplementary Figure S7a–c), we found evidence of serial mediation through IL-6 (0.004; 95 % CI: 0.001, 0.007; *p* = 0.032) but not through CRP (0.002; 95 % CI: 0.000, 0.005; *p* = 0.078) or TNF-α (0.001; 95 % CI: −0.001, 0.002; *p* = 0.330).

Third, we conducted sensitivity analyses only among participants who consistently reported a sexual minority or a heterosexual sexual orientation, excluding the 14.7 % of respondents who reported a change between a sexual minority status and a non-sexual-minority status from Waves 1 to 4. We did so given evidence that change (vs. stability) in sexual orientation identity is associated with increases in depressive symptoms (Everett, 2015); thus, the inclusion of respondents reporting a change in sexual orientation identity could inflate observed effects. However, when excluding these participants, we found a similar pattern of results across all models, including the serial mediation analyses (Supplementary Figure S8).

Fourth, to test whether our results were consistent when comparing specific sexual minority subgroups to heterosexuals, we re-ran the structural equation model including adverse childhood interpersonal events and chronically elevated inflammation as serial mediators of the association between sexual orientation and depression symptom severity separately for lesbian/gay and bisexual (vs. heterosexual) individuals (Supplementary Figures S9 and S10). Adverse childhood interpersonal events and chronic inflammation were serial mediators of the sexual orientation disparity in depression symptoms among bisexual (vs. heterosexual) individuals but not among lesbian/gay (vs. heterosexual) individuals. Specifically, results from these analyses suggested that the pattern of serial mediation observed in this study appeared to be driven by bisexuals’ disproportionate exposure to adverse childhood interpersonal events and the subsequent link to chronic inflammation among this sexual minority subgroup. This finding should be interpreted cautiously, however, as the relatively smaller sample of lesbian/gay (vs. bisexual) participants may have limited statistical power to detect subgroup differences.

Fifth, as an additional test of potential sexual minority subgroup differences, we re-ran the model of sexual orientation as a moderator of the association between inflammation and depression (i.e., test of the neuro-inflammatory sensitization hypothesis) separately for lesbian/gay and bisexual (vs. heterosexual) individuals. Results were consistent for bisexual and lesbian/gay (vs. heterosexual) respondents, indicating the lack of subgroup differences among sexual minorities in these models.

## 4. Discussion

Sexual minority young adults represent one of the highest risk groups for depression (Bostwick et al., 2010; Bränström et al., 2018; Caceres et al., 2019; Hughes et al., 2010; Plöderl and Tremblay, 2015; Semlyen et al., 2016). Applying the social signal transduction theory of depression to the context of stigma (Slavich, 2020), we used data from a longitudinal population-based cohort of sexual minority and heterosexual young adults to evaluate the role of chronically elevated inflammation in linking threats to social safety during childhood with depression symptom severity in young adulthood and in explaining the sexual orientation disparity in risk for depression symptoms. We further sought to examine evidence for an increased coupling of chronically elevated inflammation and depression symptom severity in young adulthood among sexual minorities (vs. heterosexuals), consistent with the neuro-sensitization hypothesis (Slavich and Irwin, 2014).

According to the social signal transduction theory of depression, threats to social safety during childhood can lead to chronically elevated low-grade inflammation in some individuals and increase their risk of depression in adulthood (Slavich and Sacher, 2019; Slavich and Irwin, 2014; Slavich, 2020). Our analyses supported the tenets of this theory by documenting chronically elevated levels of inflammation among young adults reporting more frequent experiences of adverse childhood interpersonal events. In line with our first hypothesis, the link between exposure to adverse childhood interpersonal events and depression symptom severity across four years of young adulthood was mediated by chronically elevated inflammation. In contrast, this indirect effect was not observed for *changes* in depression severity over this four-year period, suggesting that the associations among adverse childhood interpersonal events, chronic inflammation, and depression symptoms were stable during this timeframe. Although previous studies have shown that elevated inflammation in childhood increases risk of depression onset in young adulthood (Khandaker et al., 2014), the current study is unique in following young adults across time with multiple assessments of inflammation, providing novel evidence that the association between adverse childhood interpersonal events and depression symptom severity is mediated by chronic inflammation during young adulthood.

Our second hypothesis, also informed by the social signal transduction theory of depression (Slavich, 2020; Slavich et al., 2023; Slavich and Irwin, 2014), was that chronic inflammation precipitated by early threats to social safety would explain sexual minority young adults’ increased risk of depression symptom severity compared to heterosexuals. Our results supported this hypothesis, finding that 23 % of the association between sexual orientation and depression symptom severity was explained by sexual minorities’ disproportionate exposure to adverse childhood interpersonal events and the subsequent link to chronically elevated inflammation. Our study is the first to empirically demonstrate a role of early social stress exposure and chronically elevated inflammation in explaining sexual orientation disparities in depression symptom severity. These results cohere with previous studies showing that sexual minority individuals’ higher exposure to adverse childhood interpersonal events contributes to sexual orientation disparities in depression (McLaughlin et al., 2012; Jun et al., 2010; McCabe et al., 2022). Importantly, the present results extend these findings by identifying chronic inflammation as a mechanism through which adverse childhood interpersonal events might increase mental health risk among sexual minorities (vs. heterosexuals). At the same time, only 2.3 % of the observed effect of sexual orientation on depression symptom severity was explained via the serial indirect effect through adverse childhood interpersonal events and chronically elevated inflammation. Consequently, research is needed to identify additional mechanisms linking childhood adversity to depression symptoms among sexual minorities. As sensitivity analyses further showed that this serial indirect effect was largely driven by bisexual individuals’ increased risk of adverse childhood interpersonal events compared to heterosexuals, future work is also needed to explain why bisexual individuals may be more likely to experience early interpersonal adversity compared to their heterosexual and lesbian/gay peers.

Finally, based on the neuro-inflammatory sensitization hypothesis that individuals who are exposed to early threats to social safety experience a stronger association between inflammation and depression symptoms (Slavich and Irwin, 2014), we hypothesized a stronger link between chronically elevated inflammation and depression symptom severity among sexual minority individuals given their increased exposure to early threats to social safety as compared to their heterosexual peers (McLaughlin et al., 2012; McCabe et al., 2022). Providing partial support for this hypothesis, we found that sexual minority individuals were more likely to experience depression symptoms that co-occur with inflammation than were heterosexuals. Although previous theory and research suggest this increased coupling between inflammation and depression among sexual minorities (relative to heterosexuals) may be due to social safety threats (Diamond et al., 2021), we did not find evidence that sexual orientation moderated the full mediation model (i.e., adverse childhood events → chronic inflammation → depressive symptom severity). We suspect that this null finding is likely due to the increased statistical power required in moderated mediation models. Alternatively, the lack of moderated mediation could reflect the fact that this model only tested one of what are likely many forms of early or ongoing social threats that could underlie the stronger link between inflammation and depressive symptom severity observed among sexual minorities (vs. heterosexuals). Both of these possibilities should be tested in future studies with larger sample sizes.

Across each of these three sets of analyses, we used a latent measure of chronically elevated inflammation combining three inflammatory biomarkers rather than a single marker of inflammation from one time point, an approach that may help address inconsistencies in prior work. While a small number of studies have shown that sexual minorities experience increased levels of inflammation compared to heterosexuals (Wardecker et al., 2021; Simenson et al., 2020), others have found no such pattern (Mays et al., 2018) or mixed results depending on sexual minority subgroup (Hatzenbuehler et al., 2013; Doyle and Molix, 2016). All of these studies, however, have measured different types of inflammatory markers at a single time point (Diamond et al., 2021). The importance of measuring chronic rather than one-time markers of inflammation is underscored in our study by the lack of sexual orientation differences found in the markers of inflammation measured at one time point but the presence of significant sexual orientation differences in chronically elevated inflammation measured across two time points using multiple indicators of inflammation. Our approach also highlights the importance of examining variability in exposure to social stressors in explaining chronic elevations in inflammation based on sexual orientation. Many prior studies (Hatzenbuehler et al., 2013; Mays et al., 2018; Doyle and Molix, 2016) have assumed sexual orientation differences in stress exposure in searching for sexual orientation differences in inflammation. By actually measuring such differences, the present study finds that sexual minorities’ disproportionate exposure to adverse interpersonal events is prospectively linked to chronically elevated inflammation, which is in turn associated with their increased risk for depression in young adulthood relative to heterosexuals.

Our study points to several future directions. The present results demonstrate that chronically elevated inflammation is linked to more severe depression symptoms across a four-year period of young adulthood, supporting a key tenet of the social signal transduction theory of depression (Slavich, 2020). However, future research is needed to identify the psychological processes elicited by threats to social safety—such as hypervigilance (Hollinsaid et al., 2023)—and to evaluate their subsequent effects on chronic inflammation and depression risk. In line with the broader social safety theory, future studies can additionally explore chronically elevated inflammation linked to adverse childhood interpersonal events as a contributor to other health conditions with links to both depression and inflammation (Slavich, 2020; Slavich et al., 2023; Slavich, 2022), such as rheumatoid arthritis, chronic pain, obesity, diabetes, and cardiovascular disease, which are also elevated among sexual minority individuals compared to heterosexuals (Diamond et al., 2021; Lick et al., 2013). In addition, the social safety theory highlights the importance of examining the health consequences not only of threats to social safety as measured here, but also of the presence of social safety (e.g., affirmation, support, inclusion) (Diamond et al., 2021; Slavich et al., 2023). Accordingly, future studies should assess both factors that promote social safety and that constitute social threats.

Our results also suggest several potential targets for addressing sexual orientation disparities in depression symptoms among young adults. Interventions are needed to prevent sexual minority youths’ early and ongoing experiences of threats to social safety, such as bullying (Earnshaw et al., 2016), discrimination (Hatzenbuehler and Pachankis, 2016), and parental non-acceptance (Diamond and Aspinwall, 2003; Diamond et al., 2022). Further, psychological interventions are needed that support sexual minority individuals’ resilience in coping with such threats. Studies have identified several potential targets for such interventions, including those that improve emotion regulation skills and reduce social isolation and hopelessness (Hatzenbuehler et al., 2009; McLaughlin et al., 2009; Pachankis, 2018; Chaudoir et al., 2017). Additionally, because inflammation functions as a mechanism through which exposure to adverse childhood interpersonal events might lead to sexual orientation disparities in depression symptoms, future intervention research might find benefit in targeting inflammation directly.

This study has several methodological strengths, including its use of a large national prospective cohort recruited from a population-based sample and multiple assessments of depression symptoms and inflammatory biomarkers. At the same time, the present findings should be considered alongside study limitations. First, we relied on retrospective reports of exposure to adverse childhood interpersonal events, which could introduce recall bias and same-source reporting bias, thereby yielding an overestimate of the association between adverse childhood interpersonal events and depression symptoms. However, current experiences of depressed mood do not lead to inflated associations between self-reported depression and childhood events (Brewin et al., 1993; Hardt and Rutter, 2004); further, over-reporting of adverse childhood events is less common than under-reporting of such events in retrospective assessments (Hardt and Rutter, 2004). Second, this study prospectively followed individuals during four years of young adulthood rather than childhood and adolescence, the developmental period during which the sexual orientation disparity in depression first emerges (Irish et al., 2019; La Roi et al., 2016; Pachankis et al., 2022). As such, we were unable to test whether exposure to adverse interpersonal events in childhood increased risk of inflammation and depression in young adulthood by elevating chronic stress exposure over the full course of these developmental periods. To enable a fuller exploration of the impact of early and ongoing stress exposure, both forms of stress should be assessed in future prospective studies following individuals from childhood into adulthood. Third, our measure of adverse childhood interpersonal events focused on multiple types of interpersonal violence. A broader assessment of adverse childhood experiences, including non-social events such as natural disasters as well as identity-related social stressors such as family rejection and discrimination related to sexual minority status, could provide a fuller picture of the early experiences to which sexual minority individuals are disproportionately exposed that might also be relevant to their elevated depression risk as compared to heterosexuals.

In summary, we provide one of the first longitudinal, population-based studies to demonstrate the role of chronically elevated inflammation in linking threats to social safety during childhood with depression symptom severity in young adulthood, consistent with the social signal transduction theory of depression (Slavich, 2020). We extend this theory to the population level by finding that sexual minority young adults experience a greater risk of depression symptom severity relative to their heterosexual peers, at least in part because of their greater exposure to adverse interpersonal events in childhood and the consequent link to chronically elevated inflammation. These findings provide the strongest evidence to date for early exposure to social threats—and the subsequent effects of such threats on chronic inflammation—as dual factors that might explain sexual orientation disparities in depression symptoms, highlighting potential biopsychosocial intervention targets.

## Supplementary Material

Supplementary Data

## Figures and Tables

**Table 1:** Demographic characteristics of the PLUS cohort study sample included in the current analyses by sexual orientation (*n* = 595).

	Heterosexual *n* = 262		Sexual minority *n* = 333		Sig.
	*n*	%	*n*	%	
**Sexual orientation identity**					–
Heterosexual	262	(100.0)	0	(0.0)	
Lesbian/gay	0	(0.0)	71	(21.3)	
Bisexual or pansexual	0	(0.0)	194	(58.3)	
Other sexual orientation	0	(0.0)	19	(5.7)	
Sexual minority identity at one or more, but not all, assessment timepoints	0	(0.0)	49	(14.7)	
**Mean age - years (SD)**	26.3	(5.1)	25.3	(4.8)	*p* =.002
**Sex assigned at birth**					*p* <.001
Male	86	(32.8)	63	(18.9)	
Female	176	(67.2)	270	(81.1)	
**Gender identity**					*p* <.001
Man (cisgender and transgender)	86	(32.8)	62	(18.6)	
Woman (cisgender and transgender)	176	(67.2)	258	(77.5)	
Gender non-conforming, gender queer, or other gender	0	(0.0)	13	(3.9)	
**Education**					*p* <.001
Less than university degree	146	(55.7)	228	(68.5)	
University degree	116	(44.3)	105	(31.5)	
**Country of birth**					*p* =.354
Sweden	243	(92.7)	315	(94.6)	
Born outside of Sweden	19	(7.3)	18	(5.4)	

**Table 2:** Depression symptoms, adverse childhood interpersonal events, and biomarkers of inflammation (i.e., C-Reactive Protein [CRP], Interleukin 6 [Il-6], and Tumor Necrosis Factor alpha [TNF-α]) by sexual orientation.

	Sexual orientation			
	Heterosexual	Sexual minority	Sig.	Effect size
	Mean (SD)	Mean (SD)		Cohen’s *d* (95 % CI)
**Self-report measures**				
Depression symptoms at Wave 1	14.48 (10.86)	21.56 (12.28)	*p* <.001	0.61 (0.44, 0.77)
Depression symptoms at Wave 2	13.91 (10.19)	20.76 (11.97)	*p* <.001	0.61 (0.44, 0.76)
Depression symptoms at Wave 3	13.71 (9.94)	19.83 (11.66)	*p* <.001	0.56 (0.39, 0.73)
Depression symptoms at Wave 4	13.26 (9.67)	19.60 (11.60)	*p* <.001	0.59 (0.41, 0.76)
Adverse childhood interpersonal events	2.09 (1.79)	2.64 (2.12)	*p* <.001	0.28 (0.10, 0.45)
**Biomarkers of inflammation**				
CRP at Wave 1; mg/L	1.37 (4.84)	1.33 (1.93)	*p* =.909	−0.01 (−0.19, 0.16)
Il-6 at Wave 1; pg/mL	0.42 (0.60)	0.47 (0.39)	*p* =.255	0.10 (−0.07, 0.27)
TNF-α at Wave 1; pg/mL	2.52 (0.97)	2.52 (0.66)	*p* =.987	−0.001 (−0.17, 0.17)
CRP at Wave 3; mg/L	1.54 (2.52)	1.56 (2.67)	*p* =.955	−0.001 (−0.16, 0.17)
Il-6 at Wave 3; pg/mL	0.48 (0.51)	0.51 (0.40)	*p* =.421	0.07 (−0.10, 0.24)
TNF-α at Wave 3; pg/mL	2.52 (0.56)	2.56 (0.63)	*p* =.466	0.06 (−0.10, 0.23)
**Chronically elevated inflammation across Waves 1 and 3**				
CRP across waves	3.02 (1.89)	3.39 (1.86)	*p* =.032	0.20 (0.02, 0.38)
Il-6 across waves	2.72 (1.59)	3.01 (1.71)	*p* =.049	0.17 (0.001, 0.34)
TNF-α across waves	3.00 (1.90)	3.03 (2.00)	*p* =.861	0.02 (−0.16, 0.19)

*Note*: CI = Confidence interval.

## Data Availability

Data will be made available on request.

## Appendix A. Supplementary data

Supplementary data to this article can be found online at https://doi.org/10.1016/j.bbi.2024.03.036.
