# Supplementary material for: Threats to social safety and neuro-inflammatory mechanisms underlying sexual orientation disparities in depression symptom severity: A prospective cohort study of young adults
Source: Brain Behav Immun. Author manuscript; Available in PMC 2026 Aug 19. (PMC13486888; doi:10.1016/j.bbi.2024.03.036)
Supplement: Supplementary file 1 [file NIHMS2203710-supplement-Supplementary_Data.docx]

**Online Supplement**

**Table S1.** Correlations among depression symptoms, biomarkers of chronically elevated inflammation, adverse childhood interpersonal events, and sociodemographic variables.

**Table S2.** Chronically elevated inflammation as a mediator of the longitudinal association between adverse childhood interpersonal events and depression symptoms.

**Table S3.** Adverse childhood interpersonal events and chronically elevated inflammation as mediators of the longitudinal association between sexual orientation and depression symptoms.

**Figure S1.** Illustration of the timing of PLUS cohort assessments and measures.

**Figure S2.** Chronically elevated inflammation as a mediator of the longitudinal association between adverse childhood interpersonal events and depression symptoms adjusted for sociodemographic covariates, with depression modelled using latent growth analysis.

**Figure S3.** Adverse childhood interpersonal events and chronically elevated inflammation as mediators of the longitudinal association between sexual orientation and depression symptoms adjusted for sociodemographic covariates, with depression modelled using latent growth analysis.

**Figure S4.** Chronically elevated inflammation as a mediator of the longitudinal association between adverse childhood interpersonal events and depression symptoms adjusted for weight status and tobacco use.

**Figure S5.** Adverse childhood interpersonal events and chronically elevated inflammation as mediators of the longitudinal association between sexual orientation and depression symptoms adjusted for weight status and tobacco use.

**Figure S6a-c.** CRP across Waves 1 and 3 (Panel A), IL-6 across Waves 1 and 3 (Panel B), and TNF-α across Waves 1 and 3 (Panel C) as mediators of the longitudinal association between sexual orientation and depression symptoms.

**Figure S7a-c.** Adverse childhood interpersonal events and CRP across Waves 1 and 3 (Panel A), IL-6 across Waves 1 and 3 (Panel B), and TNF-α across Waves 1 and 3 (Panel C) as mediators of the longitudinal association between sexual orientation and depression symptoms.

**Figure S8.** Adverse childhood interpersonal events and chronically elevated inflammation as mediators of the longitudinal association between sexual orientation and depression symptoms adjusted for sociodemographic covariates among respondents consistently reporting a sexual minority or heterosexual sexual orientation.

**Figure S9.** Adverse childhood interpersonal events and chronically elevated inflammation as mediators of the longitudinal association between lesbian/gay vs. heterosexual sexual orientation and depression symptoms.

**Figure S10.** Adverse childhood interpersonal events and chronically elevated inflammation as mediators of the longitudinal association between bisexual vs. heterosexual sexual orientation and depression symptoms.

**Table S1.** Correlations among depression symptoms, biomarkers of chronically elevated inflammation, adverse childhood interpersonal events, and sociodemographic variables.

| **Variable** | **1** | **2** | **3** | **4** | **5** | **6** | **7** | **8** | **9** | **10** | **11** | **12** | **13** | **14** |
| --- | --- | --- | --- | --- | --- | --- | --- | --- | --- | --- | --- | --- | --- | --- |
| 1. Depression symptoms (W1) | - |  |  |  |  |  |  |  |  |  |  |  |  |  |
| 2. Depression symptoms (W2) | .68*** | - |  |  |  |  |  |  |  |  |  |  |  |  |
| 3. Depression symptoms (W3) | .64*** | .66*** | - |  |  |  |  |  |  |  |  |  |  |  |
| 4. Depression symptoms (W4) | .58*** | .63*** | .75*** | - |  |  |  |  |  |  |  |  |  |  |
| 5. Adverse childhood interpersonal events | .29*** | .27*** | .30*** | .32*** | - |  |  |  |  |  |  |  |  |  |
| 6. Chronically elevated CRP | .18*** | .18*** | .21*** | .19*** | .12* | - |  |  |  |  |  |  |  |  |
| 7. Chronically elevated Il-6 | .20*** | .21*** | .20*** | .22*** | .17*** | .42*** | - |  |  |  |  |  |  |  |
| 8. Chronically elevated TNF-α | .06 | .10* | .08 | .06 | .06 | .21*** | .24*** | - |  |  |  |  |  |  |
| 9. Sexual orientation (heterosexuals = 0; sexual minorities = 1) | .29*** | .31*** | .27*** | .29*** | .14*** | .10* | .08 | .004 | - |  |  |  |  |  |
| 10. Age | -.16*** | -.16*** | -.18*** | -.17*** | .02 | -.02 | .03 | .08 | -.14*** | - |  |  |  |  |
| 11. Sex-assigned at birth (male = 0; female = 1) | .16*** | .18*** | .15*** | .13*** | -.04 | .18*** | .06 | -.12** | .18*** | -.16*** | - |  |  |  |
| 12. Gender identity 1 (man = 0; woman = 1) | .17*** | .20*** | .16*** | .15*** | -.04 | .19*** | .08 | -.10** | .18*** | -.16*** | .97*** | - |  |  |
| 13. Gender identity 2 (binary = 0; gender non-conforming, gender queer, or other gender = 1) | .12** | .12** | .12** | .15*** | .05 | .04 | .04 | -.07 | .13** | -.08 | .06 | - | - |  |
| 14. Education (less than university degree = 0; university degree = 1) | -.18*** | -.23*** | -.20*** | -.23*** | -.10* | -.08 | -.02 | .06 | .12** | .44** | -.05 | -.06 | -.02 | - |
| 15. Country of birth (born in Sweden = 0; born outside of Sweden = 1) | -.03 | .02 | .06 | .06 | .06 | .02 | -.04 | -.04 | -.04 | .06 | -.04 | -.03 | -.04 | .05 |
| * Significant at *p* < .05 ** Significant at *p* < .01 *** Significant at *p* < .001. | | | | | | | | | | | | | | |

**Table S2.** Chronically elevated inflammation as a mediator of the longitudinal association between adverse childhood interpersonal events and depression symptoms.

|  | Standardized estimates with 95% confidence intervals | Sig. |
| --- | --- | --- |
| **Indirect effects of chronically elevated inflammation as a mediator:** |  |  |
| Adverse childhood interpersonal events → Chronically elevated inflammation → Depression severity across W1 to W4 | .050 (.014, .086) | .006 |
| **Direct effect on depression symptoms adjusted for chronically elevated inflammation:** |  |  |
| Adverse childhood interpersonal events → Depression severity across W1 to W4 | .266 (.185, .347) | <.001 |
| Age → Depression severity across W1 to W4 | -.116 (-.202, -.031) | .008 |
| Sex assigned at birth → Depression severity across W1 to W4 | .105 (.024, .186) | .011 |
| Education → Depression severity across W1 to W4 | -.123 (-.206, -.040) | .004 |
| Sexual orientation → Depression severity across W1 to W4 | .232 (.156, .308) | <.001 |
| Symptoms of ongoing infection at Wave 1 → Depression severity across W1 to W4 | -.017 (-.092, .059) | .661 |
| Symptoms of ongoing infection at Wave 3 → Depression severity across W1 to W4 | -.047 (-.122, .029) | .230 |
| Anti-inflammatory medication at Wave 1 → Depression severity across W1 to W4 | -.012 (-.088, .063) | .753 |
| Anti-inflammatory medication at Wave 3 → Depression severity across W1 to W4 | .009 (-.068, .085) | .827 |
| Use of contraceptives at Wave 1 → Depression severity across W1 to W4 | -.074 (-.149, .001) | .050 |
| Use of contraceptives at Wave 3 → Depression severity across W1 to W4 | .010 (-.067, .087) | .791 |
| Pregnancy at Wave 1 → Depression severity across W1 to W4 | .021 (-.079, .122) | .677 |
| Pregnancy at Wave 3 → Depression severity across W1 to W4 | -.024 (-.0126, .078) | .644 |
| **Direct effect on chronically elevated inflammation:** |  |  |
| Adverse childhood interpersonal events → Chronically elevated inflammation | .206 (.092, .319) | <.001 |
| Age → Chronically elevated inflammation | .095 (-.023, .214) | .059 |
| Sex assigned at birth → Chronically elevated inflammation | .106 (-.007, .222) | .004 |
| Education → Chronically elevated inflammation | -.056 (-.171, .060) | .278 |
| Sexual orientation → Chronically elevated inflammation | .092 (-.015, .198) | .092 |
| Symptoms of ongoing infection at Wave 1 → Chronically elevated inflammation | .021 (-.087, .128) | .707 |
| Symptoms of ongoing infection at Wave 3 → Chronically elevated inflammation | -.019 (-.126, .088) | .725 |
| Anti-inflammatory medication at Wave 1 → Chronically elevated inflammation | .066 (-.040, .171) | .224 |
| Anti-inflammatory medication at Wave 3 → Chronically elevated inflammation | .088 (-.015, .192) | .094 |
| Use of contraceptives at Wave 1 → Chronically elevated inflammation | .010 (-.090, .111) | .840 |
| Use of contraceptives at Wave 3 → Chronically elevated inflammation | .107 (.002, .211) | .046 |
| Pregnancy at Wave 1 → Chronically elevated inflammation | .004 (-.136, .144) | .955 |
| Pregnancy at Wave 3 → Chronically elevated inflammation | .117 (-.025, .258) | .106 |
|  | | |

**Table S3.** Adverse childhood interpersonal events and chronically elevated inflammation as mediators of the longitudinal association between sexual orientation and depression symptoms.

|  | Standardized estimates with 95% confidence intervals | Sig. |
| --- | --- | --- |
| **Indirect effects of adverse childhood interpersonal events and chronically elevated inflammation as a mediator:** |  |  |
| Sexual minorities vs. heterosexuals → Adverse childhood interpersonal events → Chronically elevated inflammation → Depression severity across W1 to W4 | .007 (.001, .014) | .032 |
| Sexual minorities vs. heterosexuals → Adverse childhood interpersonal events → Depression severity across W1 to W4 | .040 (.014, .065) | .002 |
| Sexual minorities vs. heterosexuals → Chronically elevated inflammation → Depression severity across W1 to W4 | .022 (-.005, .050) | .115 |
| **Direct effect on depression symptoms adjusted for adverse childhood interpersonal events and chronically elevated inflammation:** |  |  |
| Sexual minorities vs. heterosexuals → Depression severity across W1 to W4 | .232 (.156, .308) | <.001 |
| Age → Depression severity across W1 to W4 | -.116 (-.202, -.031) | .008 |
| Sex assigned at birth → Depression severity across W1 to W4 | .105 (.024, .186) | .011 |
| Education → Depression severity across W1 to W4 | -.123 (-.206, -.040) | .004 |
| Symptoms of ongoing infection at Wave 1 → Depression severity across W1 to W4 | -.017 (-.092, .059) | .661 |
| Symptoms of ongoing infection at Wave 3 → Depression severity across W1 to W4 | -.047 (-.122, .029) | .230 |
| Anti-inflammatory medication at Wave 1 → Depression severity across W1 to W4 | -.012 (-.088, .063) | .752 |
| Anti-inflammatory medication at Wave 3 → Depression severity across W1 to W4 | .009 (-.068, .085) | .827 |
| Use of contraceptives at Wave 1 → Depression severity across W1 to W4 | -.074 (-.149, .001) | .050 |
| Use of contraceptives at Wave 3 → Depression severity across W1 to W4 | .010 (-.067, .088) | .791 |
| Pregnancy at Wave 1 → Depression severity across W1 to W4 | .021 (-.079, .122) | .677 |
| Pregnancy at Wave 3 → Depression severity across W1 to W4 | -.024 (-.126, .078) | .644 |
| **Direct effect on chronically elevated inflammation:** |  |  |
| Adverse childhood interpersonal events → Chronically elevated inflammation | .206 (.092, .319) | <.001 |
| Sexual minorities vs. heterosexuals → Chronically elevated inflammation | .092 (-.023, .214) | .092 |
| Age → Chronically elevated inflammation | .096 (-.015, .198) | .114 |
| Sex assigned at birth → Chronically elevated inflammation | .106 (-.007, .220) | .067 |
| Education → Chronically elevated inflammation | -.056 (-.171, .060) | .343 |
| Symptoms of ongoing infection at Wave 1 → Chronically elevated inflammation | .021 (-.087, .128) | .707 |
| Symptoms of ongoing infection at Wave 3 → Chronically elevated inflammation | -.019 (-.126, .088) | .725 |
| Anti-inflammatory medication at Wave 1 → Chronically elevated inflammation | .066 (-.040, .171) | .224 |
| Anti-inflammatory medication at Wave 3 → Chronically elevated inflammation | .088 (-.015, .192) | .094 |
| Use of contraceptives at Wave 1 → Chronically elevated inflammation | .010 (-.090, .111) | .840 |
| Use of contraceptives at Wave 3 → Chronically elevated inflammation | .107 (.002, .211) | .046 |
| Pregnancy at Wave 1 → Chronically elevated inflammation | .004 (-.136, .144) | .954 |
| Pregnancy at Wave 3 → Chronically elevated inflammation | .117 (-.002, .258) | .106 |
| **Direct effect on adverse childhood interpersonal events:** |  |  |
| Sexual minorities vs. heterosexuals → Adverse childhood interpersonal events | .149 (.064, .234) | <.001 |
| Age → Adverse childhood interpersonal events | .098 (.002, .194) | .045 |
| Sex assigned at birth → Adverse childhood interpersonal events | -.071 (-.160, .019) | .121 |
| Education → Adverse childhood interpersonal events | -.121 (-.214, -.029) | .010 |
| Symptoms of ongoing infection at Wave 1 → Adverse childhood interpersonal events | -.010 (-.095, .075) | .811 |
| Symptoms of ongoing infection at Wave 3 → Adverse childhood interpersonal events | -.013 (-.098, .073) | .771 |
| Anti-inflammatory medication at Wave 1 → Adverse childhood interpersonal events | -.023 (-.107, .062) | .598 |
| Anti-inflammatory medication at Wave 3 → Adverse childhood interpersonal events | -.008 (-.094, .079) | .864 |
| Use of contraceptives at Wave 1 → Adverse childhood interpersonal events | .006 (-.075, .087) | .887 |
| Use of contraceptives at Wave 3 → Adverse childhood interpersonal events | -.033 (-.119, .053) | .449 |
| Pregnancy at Wave 1 → Adverse childhood interpersonal events | .054 (-.061, .170) | .357 |
| Pregnancy at Wave 3 → Adverse childhood interpersonal events | .051 (-.066, .169) | .392 |
|  | | |

**Figure S1.** Illustration of the timing of PLUS cohort assessments and measures.

**Figure S2.** Chronically elevated inflammation as a mediator of the longitudinal association between adverse childhood interpersonal events and depression symptoms adjusted for sociodemographic covariates, with depression modelled using latent growth analysis.

**Figure S3.** Adverse childhood interpersonal events and chronically elevated inflammation as mediators of the longitudinal association between sexual orientation and depression symptoms adjusted for sociodemographic covariates, with depression modelled using latent growth analysis.

**Figure S4.** Chronically elevated inflammation as a mediator of the longitudinal association between adverse childhood interpersonal events and depression symptoms adjusted for weight status and tobacco use.

**Figure S5.** Adverse interpersonal childhood events and chronically elevated inflammation as mediators of the longitudinal association between sexual orientation and depression symptoms adjusted for weight status and tobacco use.

**Figure S6a-c.** CRP across Waves 1 and 3 (Panel A), IL-6 across Waves 1 and 3 (Panel B), and TNF-α across Waves 1 and 3 (Panel C) as mediators of the longitudinal association between sexual orientation and depression symptoms.

**Figure S7a-c.** Adverse childhood interpersonal events and CRP across Waves 1 and 3 (Panel A), IL-6 across Waves 1 and 3 (Panel B), and TNF-α across Waves 1 and 3 (Panel C) as mediators of the longitudinal association between sexual orientation and depression symptoms.

**Figure S8.** Adverse childhood interpersonal events and chronically elevated inflammation as mediators of the longitudinal association between sexual orientation and depression symptoms adjusted for sociodemographic covariates among respondents consistently reporting a sexual minority or heterosexual sexual orientation.

**Figure S9.** Adverse childhood interpersonal events and chronically elevated inflammation as mediators of the longitudinal association between lesbian/gay vs. heterosexual sexual orientation and depression symptoms.

**Figure S10.** Adverse childhood interpersonal events and chronically elevated inflammation as mediators of the longitudinal association between bisexual vs. heterosexual sexual orientation and depression symptoms.
